# Elastic Pressure in Inflammation of the Breast

**Published:** 1890-05

**Authors:** 


### ELASTIC PRESSURE IN INFLAMMATION OF THE BREAST.

From a study of his own observations, Dr. Horne reaches the following conclusions: (1) Mastitis is rarely seen, except in patients who have suffered from fissured or crushed nipples, and is the result of infection. (2) As a rule, the secretion of milk continues only while the natural stimulus, as nursing, or other means, is applied. (3) The secretion of milk, both in the normal and in the inflammatory state, begins to diminish when such stimulus is withdrawn and will cease entirely within a week or two. (4) In all cases of actually or prospectively inflamed breast, well-regulated pressure by means of an elastic bandage should be applied, and no attempt should be made to nurse or remove the secretion until after the entire subsidence of the inflammatory movement. The advantages of the elastic bandage over the ordinary roller are: (*a*) It is easier of application; (*b*) the pressure is more uniform; (*c*) it is not likely to slip; (*d*) it is more comfortable; (*e*) it need not be applied over the shoulders.—*Dublin Journal of Medical Science*, Nov., 1889.

